# The genome sequence of a blow fly, *Melanomya nana* (Meigen, 1826) (Diptera: Calliphoridae)

**DOI:** 10.12688/wellcomeopenres.26844.1

**Published:** 2026-06-24

**Authors:** Olga Sivell, Ryan Mitchell, Duncan Sivell, Steven Falk, Natural History Museum Genome Acquisition Lab, Darwin Tree of Life Barcoding Collective, Wellcome Sanger Institute Tree of Life Management, Samples and Laboratory team, Wellcome Sanger Institute Scientific Operations: Sequencing Operations, Wellcome Sanger Institute Tree of Life Core Informatics team, Tree of Life Core Informatics collective, Darwin Tree of Life Consortium

**Affiliations:** 1 Natural History Museum, London, England, UK; 2 Independent researcher, Sligo Town, Country Sligo, Ireland; 3 Independent researcher, Kenilworth, England, UK

**Keywords:** Melanomya nana, blow fly, genome sequence, chromosomal, Diptera

## Abstract

We present a genome assembly from an individual male *Melanomya nana* (blow fly; Arthropoda; Insecta; Diptera; Calliphoridae). The assembly contains two haplotypes with total lengths of 1 391.98 megabases and 1 424.34 megabases. Most of haplotype 1 (99.62%) is scaffolded into 5 chromosomal pseudomolecules. Haplotype 2 was assembled to scaffold level. The mitochondrial genome has also been assembled, with a length of 27.12 kilobases. This assembly was generated as part of the Darwin Tree of Life project, which produces reference genomes for eukaryotic species found in Britain and Ireland.

## Species taxonomy

Eukaryota; Opisthokonta; Metazoa; Eumetazoa; Bilateria; Protostomia; Ecdysozoa; Panarthropoda; Arthropoda; Mandibulata; Pancrustacea; Hexapoda; Insecta; Dicondylia; Pterygota; Neoptera; Endopterygota; Diptera; Brachycera; Muscomorpha; Eremoneura; Cyclorrhapha; Schizophora; Calyptratae; Oestroidea; Calliphoridae; Calliphorinae; *Melanomya*; *Melanomya nana* (Meigen, 1826) (NCBI:txid2173294).

## Background

*Melanomya nana* (Meigen, 1826) is a species from the family Calliphoridae (Diptera). It is a small fly measuring 3–4.5 mm ( Rognes, 1991). The body is black, thinly dusted, with a slender abdomen. The posterior spiracle has a characteristic flattened and elongated shape, the coxopleural streak is absent. The parafacialia and fronto-orbital plates are dusted white; the prementum is short. The lower calypter is semicircular, bare above, with its inner edge diverging from the body. Wing cell R4 + 5 is open at wing margin, the anterior edge of the wing is slightly darkened ( Draber-Mońko, 2004; Rognes, 1991; Sivell, 2021). Male genitalia and female terminalia are characteristic and have been figured by Rognes (1991). The ovipositor is similar in shape to that of *Angioneura acerba* (Meigen, 1838) ( Rognes, 1986a).

The genus *Melanomya* Rondani, 1856 is very small, with only seven currently recognised species, five of which are Nearctic and one Oriental ( Downes, 1986; Pape, 1988; Systema Dipterorum 2025). *M. nana* is the only species from this genus occurring in the Palaearctic region. It is widely distributed in Europe and is also known from Japan and Kazakhstan ( Draber-Mońko, 2004; Pape, 1988; Rognes, 1986b, 1991). In Britain the species is widespread and occurs in a broad range of habitats including grassland, woodland, coastal habitats, heath, scrub and others ( Sivell, 2021). In Britain the adults are active from May to September, in Fennoscandia and Denmark from June to September ( Rognes, 1991; Sivell, 2021).

The biology of this species and its immature stages are unknown. Rognes (1986a) suggested it may be a parasite of snails. A study on phylogenomics and larval feeding habits of Calliphoridae conducted by Beza-Beza *et al.* (2025) supports this thesis, although direct evidence is yet to be uncovered.

*Melanomya nana* was previously included in the families Rhinophoridae ( Herting, 1993; Rognes, 1986b) and Calliphoridae ( Downes, 1965; Pape, 1986; Rognes, 1986a, 1991). Rognes (1991) placed *M. nana* in the calliphorid subfamily Malanomyinae. This subfamily has been recently incorporated into the Calliphorinae ( Yan *et al.*, 2021). Further phylogenomic research conducted by Beza-Beza *et al.* (2025) supports this change.

The high-quality genome of *Melanomya nana* was sequenced from a single male (NHMUK014036738; SAMEA11025278) from Dry Sandford Pit Nature Reserve, England. The genome of *M. nana* presented here was sequenced as part of the Darwin Tree of Life Project, a collaborative effort to sequence all named eukaryotic species in the Atlantic Archipelago of Britain and Ireland. It is the first complete genome of species from genus *Melanomya* published till date. Together with other calliphorid genomes sequenced by the Darwin Tree of Life Project ( Falk *et al.*, 2021, 2023, 2024; Mitchell *et al.*, 2023a, b, 2024; Sivell *et al.*, 2023, 2025), it will aid research into the phylogeny, systematics and phylogenetics of blow flies.

## Methods

### Sample acquisition and DNA barcoding

**Figure 1.**
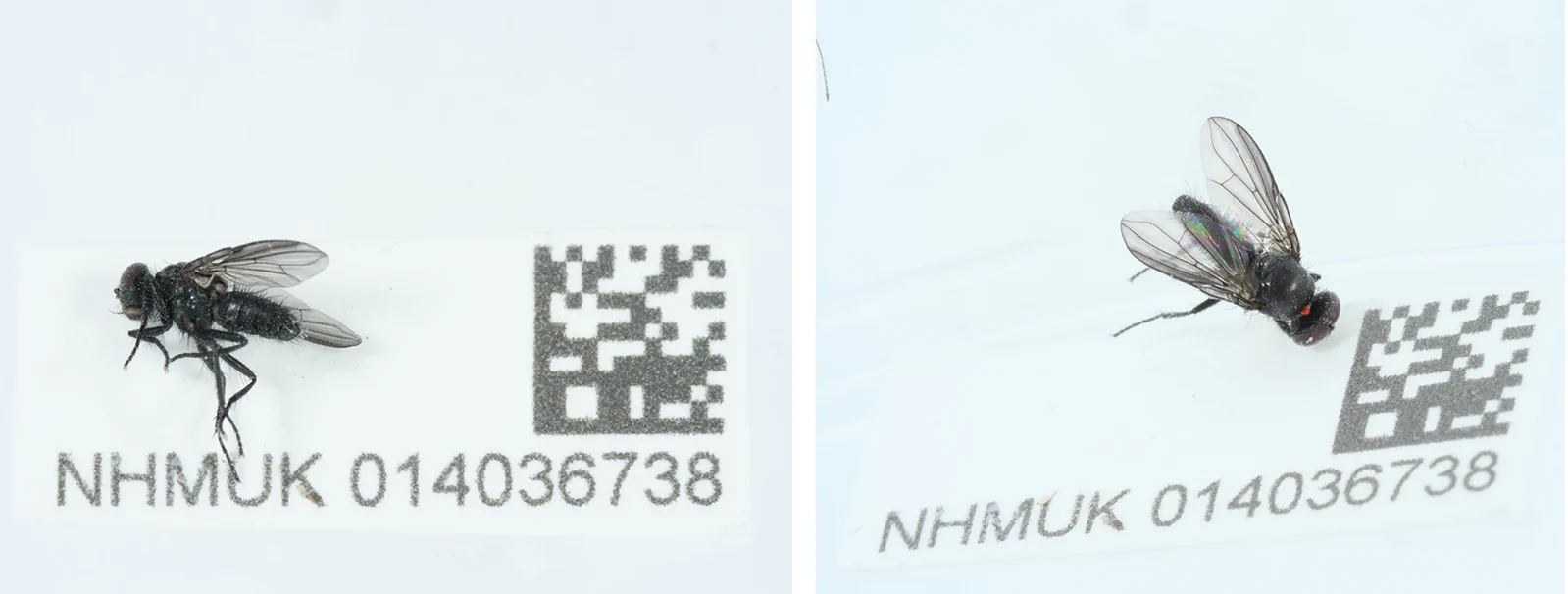
Photograph of the *Melanomya nana* (idMelNana4) specimen used for genome sequencing.

The specimen used for genome sequencing was an adult male *Melanomya nana* (specimen ID NHMUK014036738, ToLID idMelNana4; Figure 1), collected from Dry Sandford Pit, England, United Kingdom (latitude 51.6932, longitude −1.3258) on 2021-06-19. The specimen was collected by Ryan Mitchell and Olga Sivell and identified by Olga Sivell. A second specimen was used for Hi-C sequencing (specimen ID Ox000850, ToLID idMelNana2). It was collected from Wytham Woods, Oxfordshire, United Kingdom (latitude 51.769, longitude −1.339) on 2020-08-20. The specimen was collected and identified by Steven Falk.

The initial identification was verified by an additional DNA barcoding process according to the framework developed by Twyford *et al.* (2024). A small sample was dissected from the specimen and stored in ethanol, while the remaining parts were shipped on dry ice to the Wellcome Sanger Institute (WSI) (see the protocol). The tissue was lysed, the COI marker region was amplified by PCR, and amplicons were sequenced and compared to the BOLD database, confirming the species identification ( Crowley *et al.*, 2023). Following whole genome sequence generation, the relevant DNA barcode region was also used alongside the initial barcoding data for sample tracking at the WSI ( Twyford *et al.*, 2024). The standard operating procedures for Darwin Tree of Life barcoding are available on protocols.io.

### Nucleic acid extraction

Detailed protocols for nucleic acid extraction developed at the Wellcome Sanger Institute (WSI) Tree of Life Core Laboratory are available on protocols.io ( Howard *et al.*, 2025). The idMelNana4 sample was weighed and triaged to determine the appropriate extraction protocol. For HMW DNA extraction, 1.000 mg of tissue was used. The tissue was homogenised by powermashing using a PowerMasher II tissue disruptor. High molecular weight (HMW) DNA was extracted in the WSI Scientific Operations core using the Automated MagAttract v2 protocol. We used centrifuge-mediated fragmentation to produce DNA fragments in the 8–10 kb range, following the Covaris g-TUBE protocol for ultra-low input (ULI). Sheared DNA was purified by automated SPRI (solid-phase reversible immobilisation).

The concentration of the sheared and purified DNA was assessed using a Nanodrop spectrophotometer and Qubit Fluorometer using the Qubit dsDNA High Sensitivity Assay kit. Fragment size distribution was evaluated by running the sample on the FemtoPulse system. For this sample, the final post-shearing DNA had a Qubit concentration of 18.1 ng/μL and a yield of 850.70 ng, with a fragment size of 14.1 kb. The Genomic Quality Number (GQN) was 6.8.

### PacBio HiFi library preparation and sequencing

Library preparation and sequencing were performed at the WSI Scientific Operations core. Prior to library preparation, the DNA was fragmented to ~10 kb. Ultra-low-input (ULI) libraries were prepared using the PacBio SMRTbell® Express Template Prep Kit 2.0 and gDNA Sample Amplification Kit. Samples were normalised to 20 ng DNA. Single-strand overhang removal, DNA damage repair, and end-repair/A-tailing were performed according to the manufacturer’s instructions, followed by adapter ligation. A 0.85× pre-PCR clean-up was carried out with Promega ProNex beads.

The DNA was evenly divided into two aliquots for dual PCR (reactions A and B), both following the manufacturer’s protocol. A 0.85× post-PCR clean-up was performed with ProNex beads. DNA concentration was measured using a Qubit Fluorometer v4.0 (Thermo Fisher Scientific) with the Qubit HS Assay Kit, and fragment size was assessed on an Agilent Femto Pulse Automated Pulsed Field CE Instrument (Agilent Technologies) using the gDNA 55 kb BAC analysis kit. PCR reactions A and B were then pooled, ensuring a total mass of ≥500 ng in 47.4 μl.

The pooled sample underwent another round of DNA damage repair, end-repair/A-tailing, and hairpin adapter ligation. A 1× clean-up was performed with ProNex beads, followed by DNA quantification using the Qubit and fragment size analysis using the Agilent Femto Pulse. Size selection was performed on the Sage Sciences PippinHT system, with target fragment size determined by Femto Pulse analysis (typically 4–9 kb). Size-selected libraries were cleaned with 1.0× ProNex beads and normalised to 2 nM before sequencing.

The sample was sequenced on a Revio instrument (Pacific Biosciences). The prepared library was normalised to 2 nM, and 15 μL was used for making complexes. Primers were annealed and polymerases bound to generate circularised complexes, following the manufacturer’s instructions. Complexes were purified using 1.2X SMRTbell beads, then diluted to the Revio loading concentration (200–300 pM) and spiked with a Revio sequencing internal control. The sample was sequenced on a Revio 25 M SMRT cell. The SMRT Link software (Pacific Biosciences), a web-based workflow manager, was used to configure and monitor the run and to carry out primary and secondary data analysis.

### Hi-C

***Sample preparation and crosslinking***

The Hi-C sample was prepared from 20–50 mg of frozen tissue from the idMelNana2 sample using the Arima-HiC v2 kit (Arima Genomics). Following the manufacturer’s instructions, tissue was fixed and DNA crosslinked using TC buffer to a final formaldehyde concentration of 2%. The tissue was homogenised using the Diagnocine Power Masher-II. Crosslinked DNA was digested with a restriction enzyme master mix, biotinylated, and ligated. Clean-up was performed with SPRISelect beads before library preparation. DNA concentration was measured with the Qubit Fluorometer (Thermo Fisher Scientific) and Qubit HS Assay Kit. The biotinylation percentage was estimated using the Arima-HiC v2 QC beads.

***Hi-C library preparation and sequencing***

Biotinylated DNA constructs were fragmented using a Covaris E220 sonicator and size selected to 400–600 bp using SPRISelect beads. DNA was enriched with Arima-HiC v2 kit Enrichment beads. End repair, A-tailing, and adapter ligation were carried out with the NEBNext Ultra II DNA Library Prep Kit (New England Biolabs), following a modified protocol where library preparation occurs while DNA remains bound to the Enrichment beads. Library amplification was performed using KAPA HiFi HotStart mix and a custom Unique Dual Index (UDI) barcode set (Integrated DNA Technologies). Depending on sample concentration and biotinylation percentage determined at the crosslinking stage, libraries were amplified with 10–16 PCR cycles. Post-PCR clean-up was performed with SPRISelect beads. Libraries were quantified using the AccuClear Ultra High Sensitivity dsDNA Standards Assay Kit (Biotium) and a FLUOstar Omega plate reader (BMG Labtech).

Prior to sequencing, libraries were normalised to 10 ng/μL. Normalised libraries were quantified again to create equimolar and/or weighted 2.8 nM pools. Pool concentrations were checked using the Agilent 4200 TapeStation (Agilent) with High Sensitivity D500 reagents before sequencing. Sequencing was performed using paired-end 150 bp reads on the Illumina NovaSeq 6000.

### Genome assembly

Prior to assembly of the PacBio HiFi reads, a database of *k*-mer counts ( *k* = 31) was generated from the filtered reads using FastK. GenomeScope2 ( Ranallo-Benavidez *et al.*, 2020) was used to analyse the *k*-mer frequency distributions, providing estimates of genome size, heterozygosity, and repeat content.

The HiFi reads were assembled using Hifiasm in Hi-C phasing mode ( Cheng *et al.*, 2021, 2022), producing two haplotypes. Hi-C reads ( Rao *et al.*, 2014) were mapped to the primary contigs using bwa-mem2 ( Vasimuddin *et al.*, 2019). Contigs were further scaffolded with Hi-C data in YaHS ( Zhou *et al.*, 2023), using the --break option for handling potential misassemblies. The scaffolded assemblies were evaluated using Gfastats ( Formenti *et al.*, 2022), BUSCO ( Manni *et al.*, 2021) and MerquryFK ( Rhie *et al.*, 2020).

The mitochondrial genome was assembled using MitoHiFi ( Uliano-Silva *et al.*, 2023). The MitoHiFi reference was the mitochondrial genome of *Bellardia viarum* (NC_085207.1).

### Assembly curation

The assembly was decontaminated using the Assembly Screen for Cobionts and Contaminants ( ASCC) pipeline. TreeVal was used to generate the flat files and maps for use in curation. Manual curation was conducted primarily in PretextView and HiGlass ( Kerpedjiev *et al.*, 2018). Scaffolds were visually inspected and corrected as described by Howe *et al*. (2021). Manual corrections included 32 breaks and 59 joins. This reduced the scaffold count by 9.3% and increased the scaffold N50 by 1.2%. The curation process is described at https://gitlab.com/wtsi-grit/rapid-curation . PretextSnapshot was used to generate a Hi-C contact map of the final assembly.

### Assembly quality assessment

The MerquryFK tool ( Rhie *et al.*, 2020) was run in a Singularity container ( Kurtzer *et al.*, 2017) to evaluate *k*-mer completeness and assembly quality for both haplotypes using the *k*-mer database ( *k* = 31) computed prior to genome assembly. The analysis outputs included assembly QV scores and completeness statistics.

The genome was analysed using the BlobToolKit pipeline, a Nextflow implementation of the earlier Snakemake version ( Challis *et al.*, 2020). The pipeline aligns PacBio reads using minimap2 ( Li, 2018) and SAMtools ( Danecek *et al.*, 2021) to generate coverage tracks. It runs BUSCO ( Manni *et al.*, 2021) using lineages identified from the NCBI Taxonomy ( Schoch *et al.*, 2020). For the three domain-level lineages, BUSCO genes are aligned to the UniProt Reference Proteomes database ( Bateman *et al.*, 2023) using DIAMOND blastp ( Buchfink *et al.*, 2021). The genome is divided into chunks based on the density of BUSCO genes from the closest taxonomic lineage, and each chunk is aligned to the UniProt Reference Proteomes database with DIAMOND blastx. Sequences without hits are chunked using seqtk and aligned to the NT database with blastn ( Altschul *et al.*, 1990). The BlobToolKit suite consolidates all outputs into a blobdir for visualisation. The BlobToolKit pipeline was developed using nf-core tooling ( Ewels *et al.*, 2020) and MultiQC ( Ewels *et al.*, 2016), with containerisation through Docker ( Merkel, 2014) and Singularity ( Kurtzer *et al.*, 2017).

## Genome sequence report

### Sequence data

**Figure 2.**
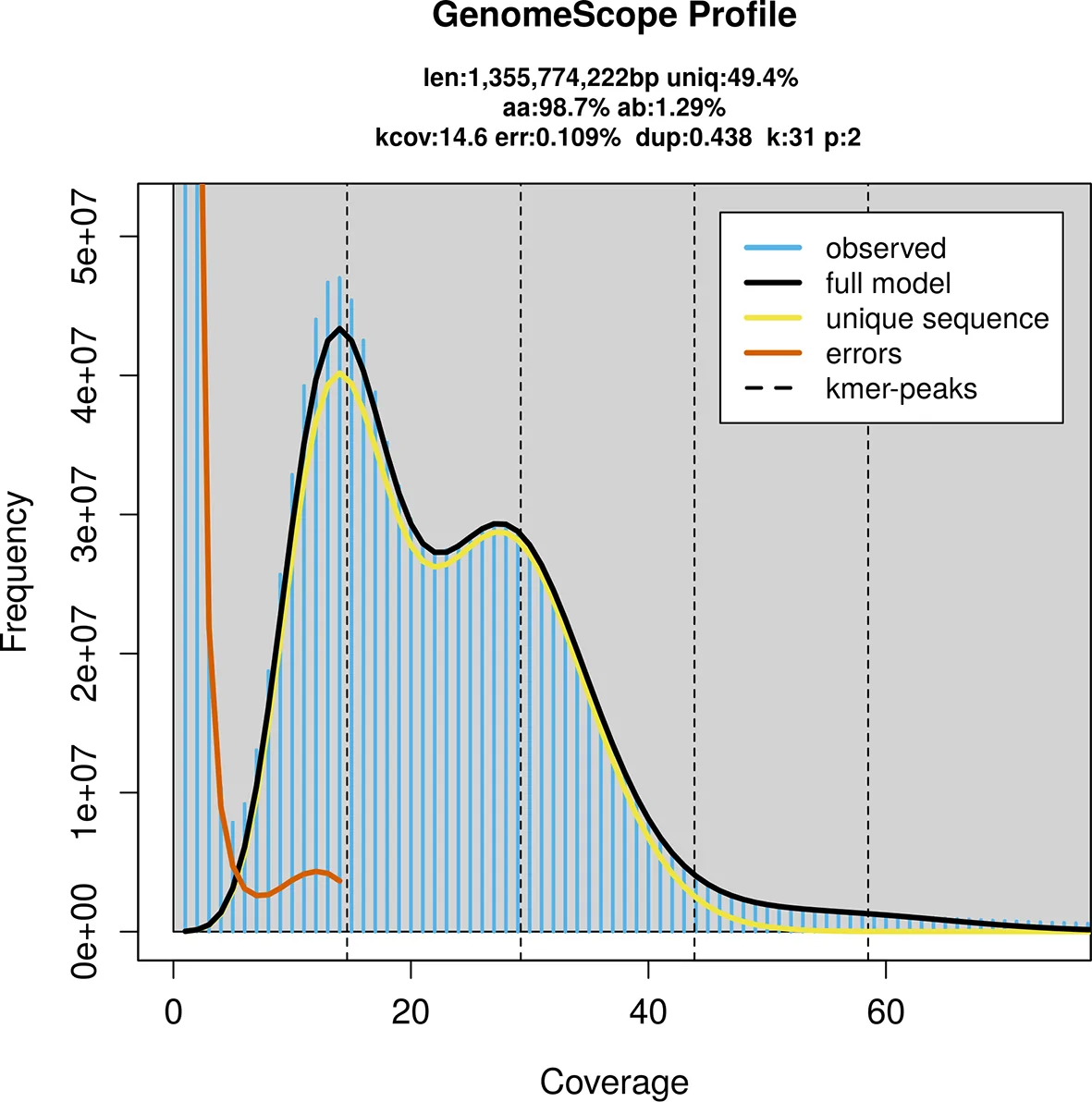
Frequency distribution of *k*-mers generated using GenomeScope2. The plot shows observed and modelled *k*-mer spectra, providing estimates of genome size, heterozygosity, and repeat content based on unassembled sequencing reads.

PacBio sequencing of the *Melanomya nana* specimen generated 41.14 Gb (gigabases) from 3.92 million reads, which were used to assemble the genome. GenomeScope2.0 analysis estimated the haploid genome size at 1 355.77 Mb, with a heterozygosity of 1.29% and repeat content of 50.98% ( Figure 2). These estimates guided expectations for the assembly. Based on the estimated genome size, the sequencing data provided approximately 29× coverage. Hi-C sequencing produced 135.30 Gb from 448.02 million reads, which were used to scaffold the assembly. Table 1 summarises the specimen and sequencing details.

**Table 1.** Specimen and sequencing data for BioProject PRJEB85046.

Platform	PacBio HiFi	Hi-C
**ToLID**	idMelNana4	idMelNana2
**Specimen ID**	NHMUK014036738	Ox000850
**BioSample (source individual)**	SAMEA11025035	SAMEA7746753
**BioSample (tissue)**	SAMEA11025278	SAMEA7746808
**Tissue**	**other somatic animal tissue**	whole organism
**Instrument**	Revio	Illumina NovaSeq 6000
**Run accessions**	ERR14209156; ERR14209155	ERR14224635
**Read count total**	3.92 million reads	448.02 million read pairs
**Base count total**	41.14 Gb	135.30 Gb

### Assembly statistics

The genome was assembled into two haplotypes using Hi-C phasing. Haplotype 1 was curated to chromosome level, while haplotype 2 was assembled to scaffold level. The final assembly has a total length of 1 391.98 Mb in 96 scaffolds, with 333 gaps, and a scaffold N50 of 251.62 Mb ( Table 2).

**Table 2.** Genome assembly statistics.

Genome assembly	Haplotype 1	Haplotype 2
**Assembly name**	idMelNana4.hap1.1	idMelNana4.hap2.1
**Assembly accession**	GCA_965605835.1	GCA_965605745.1
**Assembly level**	chromosome	scaffold
**Span (Mb)**	1 391.98	1 424.34
**Number of chromosomes**	5	-
**Number of contigs**	429	3 536
**Contig N50**	7.69 Mb	1.55 Mb
**Number of scaffolds**	96	1 895
**Scaffold N50**	251.62 Mb	248.84 Mb
**Longest scaffold length (Mb)**	352.5	345.83
**Organelles**	Mitochondrial genome: 27.12 kb	-

**Figure 3.**
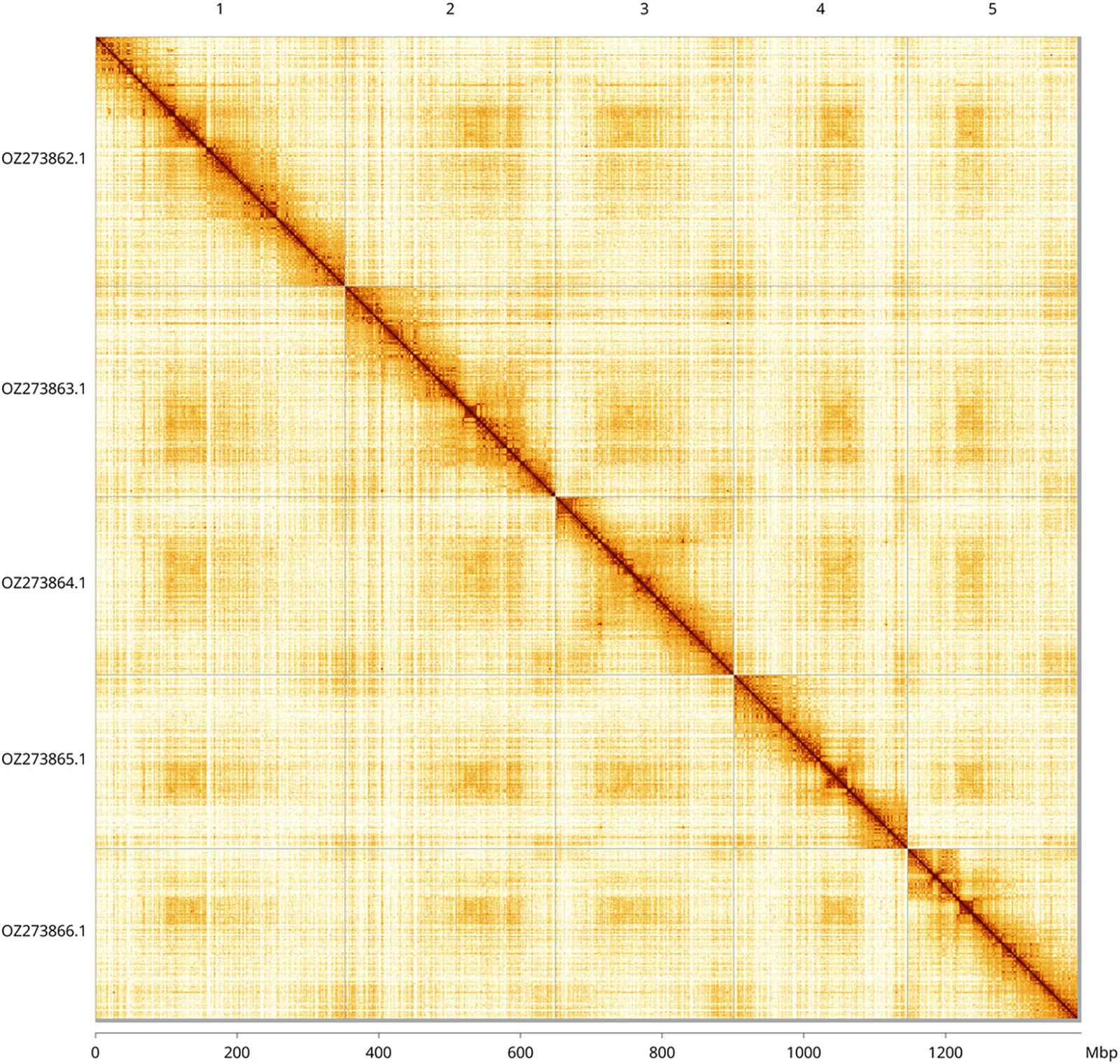
Hi-C contact map of the *Melanomya nana* genome assembly. Assembled chromosomes are shown in order of size and labelled along the axes, with a megabase scale shown below. The plot was generated using PretextSnapshot.

Most of the haplotype 1 assembly sequence (99.62%) was assigned to 5 chromosomal-level scaffolds. These chromosome-level scaffolds, confirmed by Hi-C data, are named according to size ( Figure 3; Table 3). We did not identify the sex chromosome(s) as the sex chromosomes appear to be undifferentiated in this specimen ( Vicoso & Bachtrog, 2015).

**Table 3.** Chromosomal pseudomolecules in the haplotype 1 genome assembly of *Melanomya nana* idMelNana4.

INSDC accession	Molecule	Length (Mb)	GC%
OZ273862.1	1	352.50	30.50
OZ273863.1	2	296.90	31
OZ273864.1	3	251.62	30.50
OZ273865.1	4	245.48	30.50
OZ273866.1	5	240.14	30.50

The mitochondrial genome was also assembled (length 27.12 kb, OZ273867.1). This sequence is included as a contig in the multifasta file of the genome submission and as a standalone record.

### Assembly quality metrics

**Figure 4.**
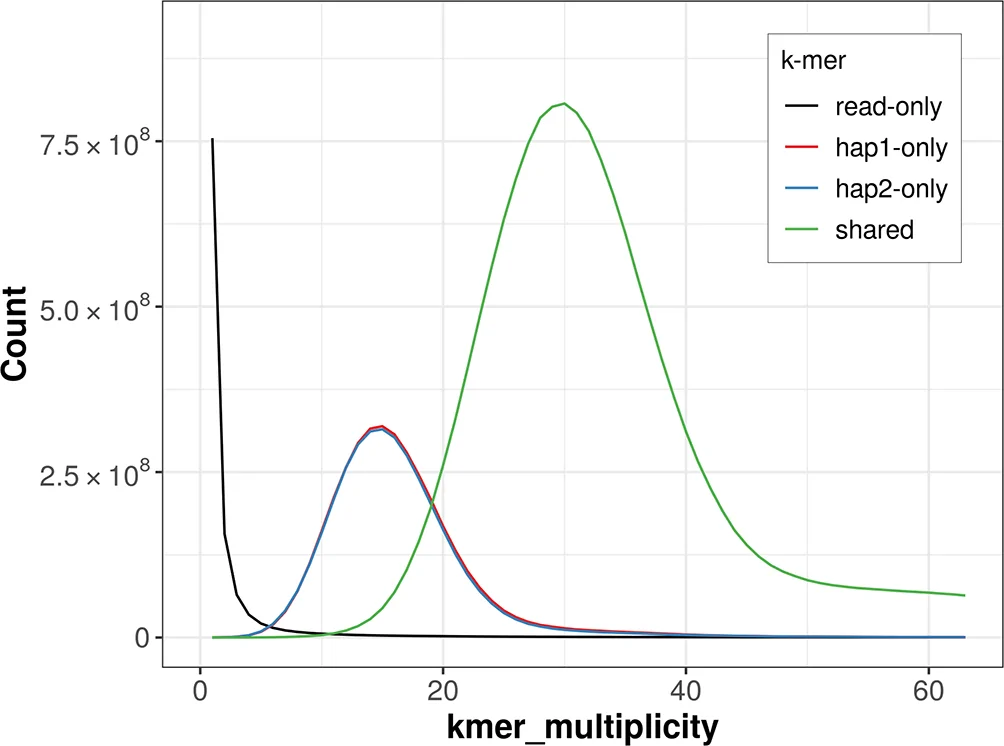
Evaluation of *k*-mer completeness using MerquryFK. This plot illustrates the recovery of *k*-mers from the original read data in the final assemblies. The horizontal axis represents *k*-mer multiplicity, and the vertical axis shows the number of *k*-mers. The black curve represents *k*-mers that appear in the reads but are not assembled. The green curve corresponds to *k*-mers shared by both haplotypes, and the red and blue curves show *k*-mers found only in one of the haplotypes.

For haplotype 1, the estimated QV is 61.1, and for haplotype 2, 61.0. When the two haplotypes are combined, the assembly achieves an estimated QV of 61.0. The *k*-mer completeness is 76.09% for haplotype 1, 75.67% for haplotype 2, and 99.09% for the combined haplotypes ( Figure 4).

**Figure 5.**
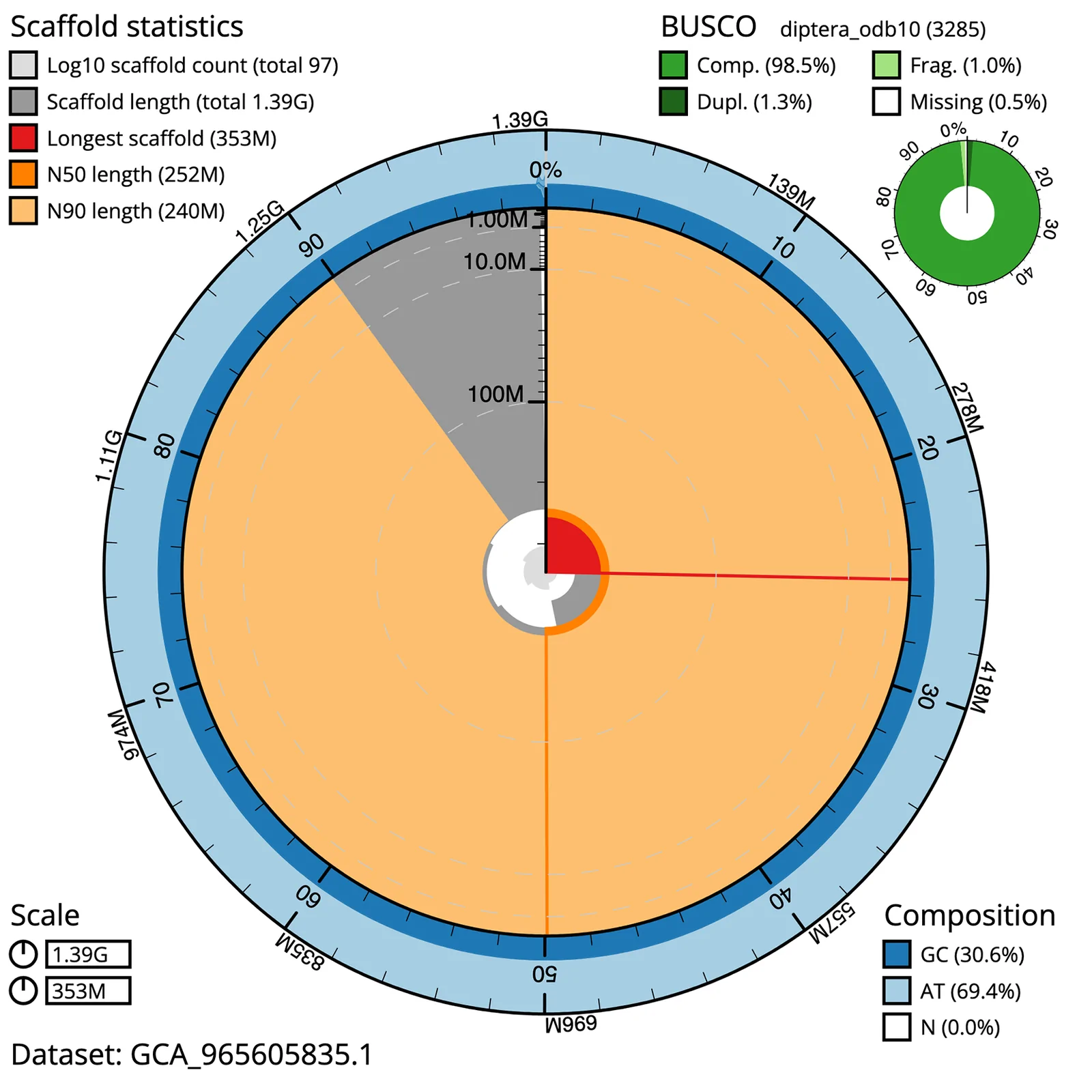
Assembly metrics for idMelNana4.hap1.1. The BlobToolKit snail plot provides an overview of assembly metrics and BUSCO gene completeness. The circumference represents the length of the whole genome sequence, and the main plot is divided into 1 000 bins around the circumference. The outermost blue tracks display the distribution of GC, AT, and N percentages across the bins. Scaffolds are arranged clockwise from longest to shortest and are depicted in dark grey. The longest scaffold is indicated by the red arc, and the deeper orange and pale orange arcs represent the N50 and N90 lengths. A light grey spiral at the centre shows the cumulative scaffold count on a logarithmic scale. A summary of complete, fragmented, duplicated, and missing BUSCO genes in the set is presented at the top right. An interactive version of this figure can be accessed on the BlobToolKit viewer.

**Figure 6.**
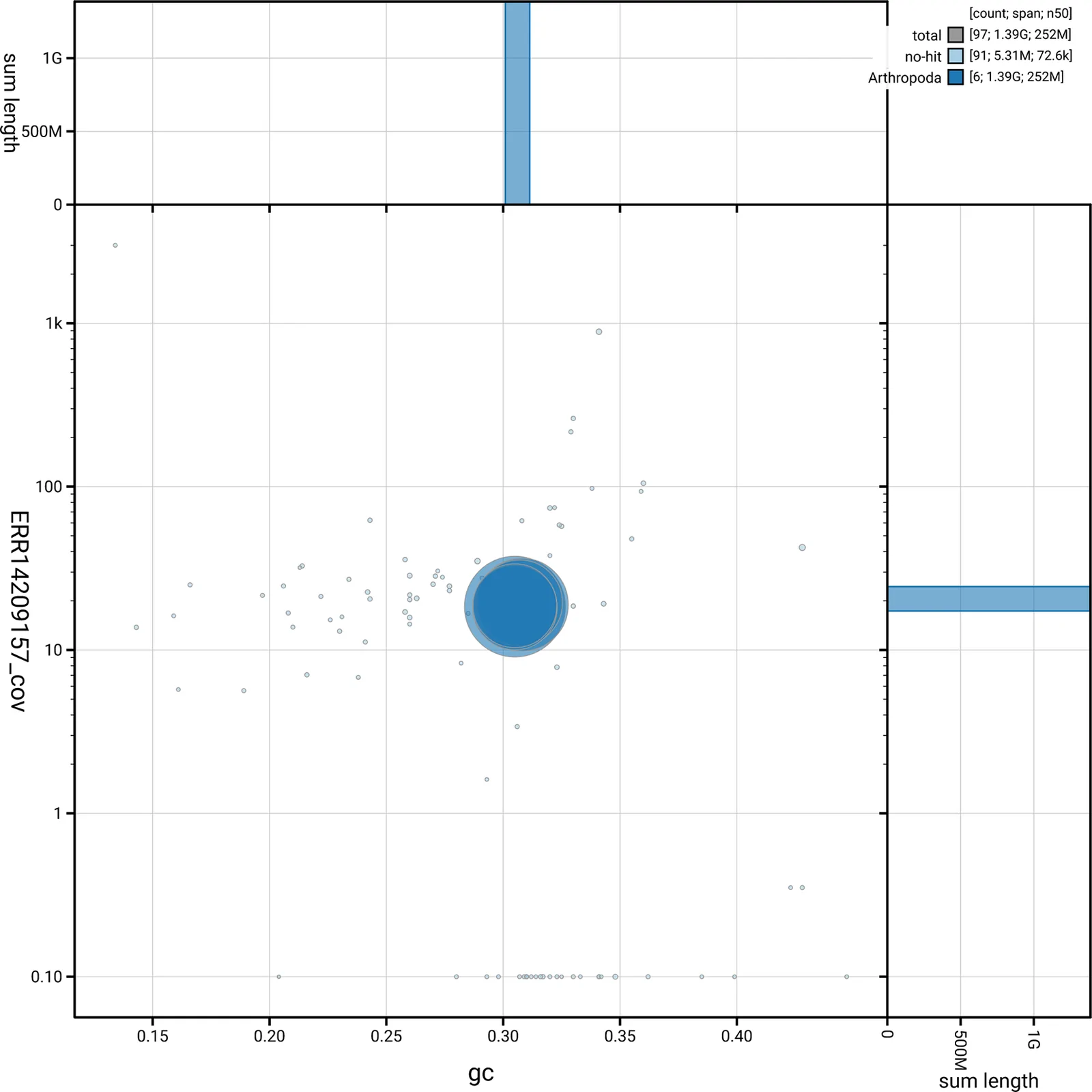
BlobToolKit blob plot for idMelNana4.hap1.1. The plot shows base coverage (vertical axis) and GC content (horizontal axis). The circles represent scaffolds, with the size proportional to scaffold length and the colour representing phylum membership. The histograms along the axes display the total length of sequences distributed across different levels of coverage and GC content. An interactive version of this figure is available on the BlobToolKit viewer.

BUSCO analysis using the diptera_odb10 reference set ( *n* = 3 285) identified 98.5% of the expected gene set (single = 97.2%, duplicated = 1.3%) in haplotype 1. For haplotype 2, BUSCO v.5.8.3 analysis identified 97.6% of the expected gene set (single = 95.9%, duplicated = 1.7%). The snail plot in Figure 5 summarises the scaffold length distribution and other assembly statistics for haplotype 1. The blob plot in Figure 6 shows the distribution of scaffolds by GC proportion and coverage for haplotype 1.

Table 4 lists the assembly metric benchmarks adapted from Rhie *et al.* (2021) and the Earth BioGenome Project Report on Assembly Standards January 2026. The EBP metric, calculated for haplotype 1, is **6.C.Q61**, meeting the recommended 6.C.Q40 reference standard.

**Table 4.** Earth Biogenome Project summary metrics for the *Melanomya nana* assembly.

Measure	Value	Benchmark
EBP summary (haplotype 1)	6.C.Q61	6.C.Q40
Contig N50 length	7.69 Mb	≥ 1 Mb
Scaffold N50 length	251.62 Mb	= chromosome N50
Consensus quality (QV)	Haplotype 1: 61.1; haplotype 2: 61.0; combined: 61.0	≥ 40
*k*-mer completeness	Haplotype 1: 76.09%; Haplotype 2: 75.67%; combined: 99.09%	≥ 95%
BUSCO	C:98.5% [S:97.2%, D:1.3%], F:1.0%, M:0.5%, n:3 285	S > 90%; D < 5%
Percentage of assembly assigned to chromosomes	99.62%	≥ 90%

Notes: The EBP summary uses log10(Contig N50); chromosome-level (C) or log10(Scaffold N50); Q (Merqury QV). BUSCO: C = complete; S = single-copy; D = duplicated; F = fragmented; M = missing; n = orthologues.

**Table 5.** Software versions and sources.

Software	Version	Source
BLAST	2.14.0	ftp://ftp.ncbi.nlm.nih.gov/blast/executables/blast+/
BlobToolKit	4.4.6	https://github.com/blobtoolkit/blobtoolkit
BUSCO	5.8.3	https://gitlab.com/ezlab/busco
bwa-mem2	2.2.1	https://github.com/bwa-mem2/bwa-mem2
DIAMOND	2.1.8	https://github.com/bbuchfink/diamond
fasta_windows	0.2.4	https://github.com/tolkit/fasta_windows
FastK	1.1	https://github.com/thegenemyers/FASTK
GenomeScope2.0	2.0.1	https://github.com/tbenavi1/genomescope2.0
Gfastats	1.3.6	https://github.com/vgl-hub/gfastats
Hifiasm	0.19.8-r603	https://github.com/chhylp123/hifiasm
HiGlass	1.13.4	https://github.com/higlass/higlass
MerquryFK	1.1.0-c1	https://github.com/thegenemyers/MERQURY.FK
Minimap2	2.28-r1209	https://github.com/lh3/minimap2
MitoHiFi	3.2.2	https://github.com/marcelauliano/MitoHiFi
MultiQC	1.14; 1.17 and 1.18	https://github.com/MultiQC/MultiQC
Nextflow	23.10.0; 24.10.4	https://github.com/nextflow-io/nextflow
PretextSnapshot	0.0.4	https://github.com/sanger-tol/PretextSnapshot
PretextView	1.0.3	https://github.com/sanger-tol/PretextView
samtools	1.17; 1.2; 1.20; 1.21	https://github.com/samtools/samtools
sanger-tol/ascc	0.1.0	https://github.com/sanger-tol/ascc
sanger-tol/blobtoolkit	v0.8.0	https://github.com/sanger-tol/blobtoolkit
sanger-tol/curationpretext	1.4.2	https://github.com/sanger-tol/curationpretext
Seqtk	1.4-r122	https://github.com/lh3/seqtk
Singularity	3.9.0	https://github.com/sylabs/singularity
TreeVal	1.2.0	https://github.com/sanger-tol/treeval
YaHS	1.2.2	https://github.com/c-zhou/yahs

## Author information

Contributors are listed at the following links:

• Members of the Natural History Museum Genome Acquisition Lab
• Members of the Darwin Tree of Life Barcoding collective
• Members of the Wellcome Sanger Institute Tree of Life Management, Samples and Laboratory team
• Members of Wellcome Sanger Institute Scientific Operations – Sequencing Operations
• Members of the Wellcome Sanger Institute Tree of Life Core Informatics team
• Members of the Tree of Life Core Informatics collective
• Members of the Darwin Tree of Life Consortium

## Wellcome Sanger Institute – Legal and Governance

The materials that have contributed to this genome note have been supplied by a Darwin Tree of Life Partner. The submission of materials by a Darwin Tree of Life Partner is subject to the **‘Darwin Tree of Life Project Sampling Code of Practice’**, which can be found in full on the Darwin Tree of Life website. By agreeing with and signing up to the Sampling Code of Practice, the Darwin Tree of Life Partner agrees they will meet the legal and ethical requirements and standards set out within this document in respect of all samples acquired for, and supplied to, the Darwin Tree of Life Project. Further, the Wellcome Sanger Institute employs a process whereby due diligence is carried out proportionate to the nature of the materials themselves, and the circumstances under which they have been/are to be collected and provided for use. The purpose of this is to address and mitigate any potential legal and/or ethical implications of receipt and use of the materials as part of the research project, and to ensure that in doing so we align with best practice wherever possible. The overarching areas of consideration are:

• Ethical review of provenance and sourcing of the material
• Legality of collection, transfer and use (national and international)

Each transfer of samples is further undertaken according to a Research Collaboration Agreement or Material Transfer Agreement entered into by the Darwin Tree of Life Partner, Genome Research Limited (operating as the Wellcome Sanger Institute), and in some circumstances, other Darwin Tree of Life collaborators.

## Data Availability

European Nucleotide Archive: Melanomya nana (little black blowfly). Accession number PRJEB85046. The genome sequence is released openly for reuse. The *Melanomya nana* genome sequencing initiative is part of the Darwin Tree of Life Project (PRJEB40665) and the Sanger Institute Tree of Life Programme (PRJEB43745). All raw sequence data and the assembly have been deposited in INSDC databases. The genome will be annotated using available RNA-Seq data and presented through the Ensembl pipeline at the European Bioinformatics Institute. Raw data and assembly accession identifiers are reported in Tables 1 and 2. Production code used in genome assembly at the WSI Tree of Life is available at https://github.com/sanger-tol . Table 5 lists software versions used in this study.
