# Supplementary material for: The genome sequence of a blow fly, Melanomya nana (Meigen, 1826) (Diptera: Calliphoridae)
Source: Wellcome Open Res. 2026 Jun 24;11:374. [Version 1] doi: 10.12688/wellcomeopenres.26844.1 (PMC13550902; doi:10.12688/wellcomeopenres.26844.1)
Supplement: Peer review history

# Reviewer report: Reviewer response for version 1

**DOI:** 10.21956/wellcomeopenres.29558.r159181

**Published:** 2026-07-11

**Authors:** Sivasankaran Kuppusamy

**Affiliations:** 1 Loyola College, Chennai, Tamil Nadu, India

Authors have sequenced and assembled the chromosome-level genome of *Melanomya nana *(Meigen, 1826). Authors have used the appropriate methods for HWM DNA isolation, SMRT library preparation, sequencing, assembling and annotations.

**Comments on the manuscript**

 The taxonomic hierarchy of the species can be deleted in the abstract and it may be replaced in the background of the manuscript.  

 Authors have assembled the mitochondrial genome in the analysis and length is 27. 12 kb. The insect mitochondrial genome size is usually 14 -19 kb in length. I observed that the mitochondrial genome of *Melanomya nana*, AT-rich or *D-loop* region is too long at the end of the sequence. I think there is an assembly error of the mitogenome. Authors should check again the mitogenome of *M. nana*. 

 Authors haven’t given the details about protein-coding genes, non-coding genes and gene transcripts. These details may be included in the table and text.  

 Overall, the research article is prepared well, and the manuscript meets the necessary scientific standards. It is suitable for indexing.

Are sufficient details of methods and materials provided to allow replication by others?

Yes

Is the rationale for creating the dataset(s) clearly described?

Yes

Are the datasets clearly presented in a useable and accessible format?

Yes

Are the protocols appropriate and is the work technically sound?

Yes

Reviewer Expertise:

Phylogenetic analysis of Noctuoidea moths using mitogenome sequence

I confirm that I have read this submission and believe that I have an appropriate level of expertise to confirm that it is of an acceptable scientific standard.

# Reviewer report: Reviewer response for version 1

**DOI:** 10.21956/wellcomeopenres.29558.r159182

**Published:** 2026-07-02

**Authors:** Jaakko Pohjoismäki

**Affiliations:** 1 University of Eastern Finland, Joensuu, Finland

The Natural History Museum Genome Acquisition Laboratory, Olga Sivell, Ryan Mitchell, Duncan Sivell, and the Darwin Tree of Life Consortium present a chromosome-level reference genome assembly for *Melanomya nana*, a small and inconspicuous blow fly (Diptera: Calliphoridae). Although the species is poorly known outside specialist circles, it represents an important addition to the growing genomic resources for oestroid flies. It is also worth noting that species such as M. nana are not obvious targets for genome sequencing, making this resource especially valuable for understanding the evolution, systematics and diversification of higher Diptera.

 The manuscript provides a particularly thorough and informative background, including an up-to-date account of the taxonomy and systematic placement of *Melanomya*.

 The genome assembly follows the established Darwin Tree of Life workflow and is of high quality despite being generated from approximately 29× sequence coverage. The final primary assembly spans 1.39 Gb in only 96 scaffolds, with 99.6% of the assembly assigned to five chromosome-level scaffolds and a scaffold N50 of 251.6 Mb. Although no sex chromosome was identified, this may reflect the biology of the species rather than an assembly limitation. Assembly quality metrics are excellent, with an estimated QV of 61.1 for the primary haplotype (61.0 for the combined assembly), combined k-mer completeness of 99.1%, and BUSCO completeness of 98.5% using the Diptera reference dataset.

 Overall, this is a well-written genome note describing a high-quality reference genome that will be a valuable resource for comparative genomics and dipteran evolutionary research. I have no substantive concerns and recommend the manuscript for indexing in its current form.

Are sufficient details of methods and materials provided to allow replication by others?

Yes

Is the rationale for creating the dataset(s) clearly described?

Yes

Are the datasets clearly presented in a useable and accessible format?

Yes

Are the protocols appropriate and is the work technically sound?

Yes

Reviewer Expertise:

Molecular biology and genetics; species diversity; biodiversity genomics; taxonomy of Diptera

I confirm that I have read this submission and believe that I have an appropriate level of expertise to confirm that it is of an acceptable scientific standard.
